# Implementation of Dropout Neuronal Units Based on Stochastic Memristive Devices in Neural Networks with High Classification Accuracy

**DOI:** 10.1002/advs.202001842

**Published:** 2020-07-26

**Authors:** He‐Ming Huang, Yu Xiao, Rui Yang, Ye‐Tian Yu, Hui‐Kai He, Zhe Wang, Xin Guo

**Affiliations:** ^1^ State Key Laboratory of Material Processing and Die and Mould Technology Laboratory of Solid State Ionics School of Materials Science and Engineering Huazhong University of Science and Technology Wuhan 430074 P. R. China

**Keywords:** artificial synapses, dropout neuronal units, memristive neural networks, sample classification, stochastic memristive devices

## Abstract

Neural networks based on memristive devices have achieved great progress recently. However, memristive synapses with nonlinearity and asymmetry seriously limit the classification accuracy. Moreover, insufficient number of training samples in many cases also have negative effect on the classification accuracy of neural networks due to overfitting. In this work, dropout neuronal units are developed based on stochastic volatile memristive devices of Ag/Ta_2_O_5_:Ag/Pt. The memristive neural network using the dropout neuronal units effectively solves the problem of overfitting and mitigates the negative effects of the nonideality of memristive synapses, eventually achieves a classification accuracy comparable to the theoretical limit. The stochastic and volatile switching performances of the Ag/Ta_2_O_5_:Ag/Pt device are attributed to the stochastical rupture of the Ag filament under high electrical stress in the Ta_2_O_5_ layer, according to the TEM observation and the kinetic Monte Carlo simulation.

## Graphical abstract

Dropout neuronal units are implemented based on stochastic volatile memristive devices of Ag/Ta_2_O_5_:Ag/Pt. With the help of dropout neuronal units, the memristive neural network achieves a high accuracy comparable to the theoretical limit in classifying handwritten digits.

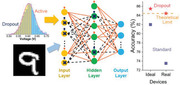

Memristive neural networks are becoming more and more efficient in the sample classification, the face recognition and the natural language processing. The integration of memristive devices with complementary metal oxide semiconductor (CMOS) devices in the Si technology further improves the performance of memristive neural networks with low power consumption.^[^ ^1^ ^]^ In memristive neural networks, synapses and neuronal units are essential elements. Thanks to the compact structure and intrinsic learning ability,^[^ ^2^, ^3^, ^4^, ^5^, ^6^, ^7^, ^8^ ^]^ and the advantages of parallel computing and low energy consumption,^[^ ^2^, ^9^, ^10^, ^11^, ^12^, ^13^, ^14^, ^15^ ^]^ memristive devices are commonly used as artificial synapses. However, the conductance (weight) of many memristive devices is modulated nonlinearly and asymmetrically during training,^[^ ^16^, ^17^, ^18^, ^19^, ^20^, ^21^ ^]^ which limits the classification accuracy.^[^ ^8^ ^]^ Great efforts were made to improve the performances of memristive devices, such as developing three‐terminal devices,^[^ ^4^ ^]^ preparing changeable compliance current devices,^[^ ^22^ ^]^ and designing specific pulses for SET/RESET.^[^ ^23^ ^]^ Based on these methods, some devices show the ability of linear and symmetrical updating.^[^ ^3^ ^]^

Owing to the versatile functions of memristive devices, such as fast volatile switching performance, neuronal units are able to be implemented with memristive devices. However, unlike the fruitful development of memristive synapses, researches about neuronal units are still in the initial stage. A neuronal unit takes charge of information processing, thus it calls for the short‐term dynamics similar to a biological neuron.^[^ ^9^, ^24^, ^25^, ^26^ ^]^ Therefore, volatile switching devices combined with simple external circuits are used to emulate some neuronal functions. For example, the volatile switching dynamics was utilized to mimic the functions of the ionic channels or the membrane potential of the Hodgkin–Huxley neuron^[^ ^24^, ^27^ ^]^ or the leaky integrate‐and‐fire neuron.^[^ ^9^, ^26^, ^28^ ^]^ Reported neuronal units focus mostly on the realization of the neuronal functions. Neural networks based on these neuronal units and nonideal synapses with nonlinearity and asymmetry show limited performances, because neuronal units have no tolerance to the nonideality of memristive synapses. To develop compact neuronal units with high tolerance to the nonideality of connected synapses is a new idea to realize high classification accuracy in memristive neural networks.

Except for the synaptic performances, there are other factors affecting the eventual performance of memristive neural networks.^[^ ^29^, ^30^, ^31^ ^]^ Overfitting usually happens in cases of a small number of training samples or in too complicated networks.^[^ ^32^ ^]^ It means that the network shows poor generalization, i.e., very high accuracy for training data but very limited ability to predict data out of the training set. This is because the network treats the noise in training samples as the feature. To prevent overfitting, several algorithms were applied, including regularization and principal component analysis (PCA). Regularization avoids too complicated network by adding a regularization parameter in the error function. Sheridan et al. obtained the regularization parameter by the external circuit to train a dictionary for sparse coding, while the sparsity was modulated by the regularization parameter.^[^ ^33^ ^]^ For the PCA approach, the noise in the input data is filtered out based on the PCA algorithm, then data without noise are fed into the neural network. The PCA algorithm was implemented in hardware by Choi et al. to improve the classification success rate for standard breast cancer screening database.^[^ ^34^ ^]^ However, these two approaches need additional external computing resources to address the overfitting issue.

Noteworthy, there exists another effective and general way to solve the problem of overfitting, i.e., to add the dropout function in neuronal units.^[^ ^32^ ^]^ In this approach, a neuronal unit drops out randomly at a fixed probability in the training process to get a thinned network, while all units are activated in the testing process. Thus, different combinations of neuronal units are activated in each training process, resulting in various thinned networks in training. Since all neuronal units are activated in testing, the final network is the average of trained thinned networks. This average process dilutes the noise from the training data, enhancing the generalization of the network in the case of insufficient number of samples. Considering that the synaptic weight updating and the dropout in training are all nonlinear, dropout neural networks are much more tolerant to the nonideality of the synaptic modulation. Therefore, the dropout function not only prevents overfitting, but also mitigates the requirements for memristive synapses.

To implement the dropout function in neuronal units, devices with stochastic dynamics are required. Several stochastic dynamics originating from thermal, temporal or spatial randomness were found in various memristive devices.^[^ ^2^, ^22^, ^28^ ^]^ For example, Kumar et al.^[^ ^35^ ^]^ found that thermal randomness provided an extra coupled parameter for the intrinsic chaotic randomness in the NbO_2_ device, and this thermal randomness helped to prevent the global synchronization in a Hopfield computing network. Jiang et al.^[^ ^36^ ^]^ reported that the temporal randomness in their Ag/Ag:SiO_2_/Pt diffusive memristors, i.e., the random switching‐delay time could be used in a true random number generator. The spatial randomness is generally related to the random geometry changes of conducting filaments, as reported in TaO*_x_*/HfO*_x_* ^[^ ^37^ ^]^ and phase change devices.^[^ ^28^ ^]^ Actually, the spatial randomness naturally exists in most of filamentary‐type memristive devices. However, the hardware implementation of neuronal units with the dropout effect has not been reported yet.

In this work, memristive devices Ag/Ta_2_O_5_:Ag/Pt with spatially stochastic dynamics are fabricated. The device shows the stochastic low resistance states and volatile resistive switching behavior. The dropout function is implemented by connecting the stochastic volatile memristor to a comparator with a threshold. Once the current of the memristive device reaches the threshold, the comparator triggers the neuronal unit stochastically. To further simplify the peripheral circuit, the Manhattan rule^[^ ^12^, ^38^ ^]^ using the identical potentiating or depressing pulses in training, is used in constructing neural networks. The accuracy of the neural network in the case of lacking training samples is significantly increased through applying the dropout neuronal units.

*Stochastic Memristive Devices*: Top view of the Ta_2_O_5_:Ag‐based memristive devices (5 × 5 µm^2^) is shown in **Figure** **1**a; each device assumes a crossbar structure. TEM image of the cross‐section of the as‐prepared device is shown in Figure 1b; clean and sharp interfaces between individual layers are clearly visible. In the 12 nm thick oxide layer, the existence of Ag additive is verified by EDX (Figure S1, Supporting Information). The atomic ratio of Ag in the Ta_2_O_5_:Ag layer is calculated to be ≈3 at% based on the XPS measurement (Figure S2 and Table S1, Supporting Information).

**Figure 1:**
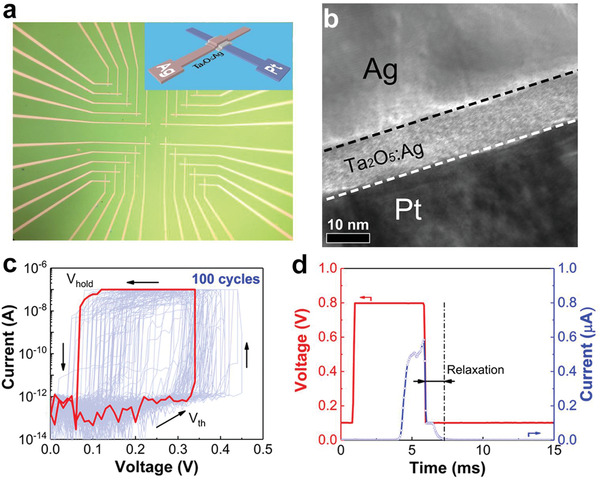
Microstructure and switching behavior of the Ag/Ta_2_O_5_:Ag/Pt memristive device. a) Optical photograph and schematic of the device; b) TEM image of the cross‐section of the device; c) current–voltage curves of the device in 100 sweep loops. When the applied voltage is across a threshold, the device switches to the LRS; once the voltage is below a hold one, the device switches from the LRS to the HRS. The threshold and hold voltages of the device are both stochastic. d) Responses of the device to electrical pulses. By applying a voltage pulse with amplitude beyond the threshold voltage, the device switches to the LRS after a delay time, then the device relaxes to its initial state in several milliseconds.

Stochastic threshold switching behavior was obtained in the Ag/Ta_2_O_5_:Ag/Pt device, as shown in Figure 1c. With a compliance current of 100 nA, the device performs a forming‐free threshold switching. Once the applied voltage reaches widely distributed thresholds, the device switches to the low resistance state (LRS); then the resistance of the device turns back to the high resistance state (HRS) quickly when the applied potential is lower than a hold voltage. The dynamic switching process was recorded by an oscilloscope. To prevent the hard breakdown, a resistor of 1 MΩ was connected to the device in series in the testing process. As shown in Figure 1d, a pulse of 0.8 V × 5 ms is applied to the device, and the current is read by a bias of 0.1 V. There is a 3.3 ms delay before the device switches to the LRS, then the device soon relaxes to its original state in 1.2 ms. This kind of volatile switching behavior is beneficial for the realization of a neuronal unit.

The LRS of the device performs stochastic feature, which is clearly shown in the evolution of the LRS current by applying a train of identical electrical pulses, as shown in **Figure** **2**a (more data are available in Figures S3 and S4 in the Supporting Information). The LRS current randomly changes even though the applied pulses are identical. To clarify the stochastic properties of the device, pulse trains, each of which was consisted of 10^3^ identical pulses, were applied on the device, and the LRS currents were recorded in real‐time. Four pulse trains with different pulse amplitudes were applied, and the probability density graph of the LRS currents are shown in Figure 2b. The LRS currents show the Gaussian‐like distribution under each pulse train with a given amplitude. By analyzing the cycle‐to‐cycle variation shown in Figure S5 (Supporting Information), more stable stochastics are found at the pulse amplitudes of 0.6 and 0.7 V. In addition, several devices were tested under the pulse of 0.7 V, demonstrating similar stochastic performances, as shown in Figure S6 (Supporting Information). To get a reliable randomness and protect the device in the endurance test, the amplitude of 0.7 V was used in the later testing.

**Figure 2:**
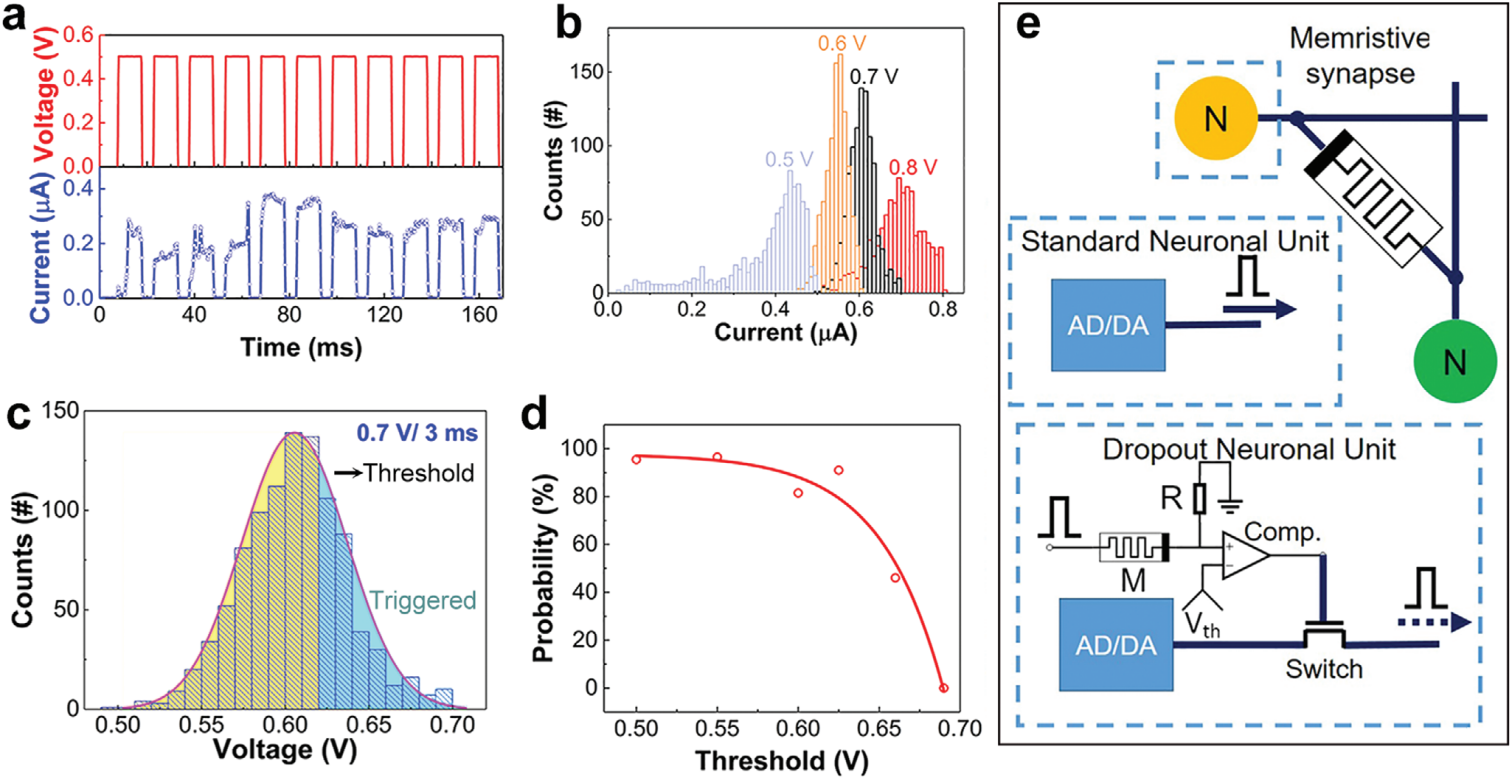
Stochastic performances of the Ag/Ta_2_O_5_:Ag/Pt device. a) Different responses of the device to ten identical electrical pulses; the response current values are random. b) Distribution of the response current values to the applied pulses with different amplitudes. c) Response currents of the device are divided into two parts by setting a threshold. d) Implementation of the probability of the neuronal unit. e) Schematic diagrams of the standard and dropout neuronal units. The AD/DA modules receive and generate spikes, and the comparator with a stochastic memristive device controls the switch, so that the neuronal unit is activated or dropped out stochastically.

The stochastics was realized in a circuit composed of a comparator with a set threshold *V*_th_ (testing circuit is shown in the low panel of Figure 2e). The stochastic device *M* is in series to a resistor *R*, and the voltage over *R* is set as the noninverting input of the comparator, while the threshold voltage is the inverting input of the comparator. Once the voltage over *R* is higher than the threshold, the output of the comparator is triggered and recorded. The voltage over the resistor *R* is determined by the LRS of the device *M*, and the stochastics is represented by the output of the comparator. For example, as shown in Figure 2c, a threshold of 0.62 V divides the LRS currents induced by applying pulses with an amplitude of 0.7 V into two parts: currents over the threshold trigger the output of the comparator, while those below the threshold cannot trigger the comparator. By this way, the probability ${\hat{p}}_{\mathrm{pulse}}$ is able to be read out by counting triggered pulses

(1) $${\hat{p}}_{\mathrm{pulse}}=\frac{n_{\mathrm{triggered}}}{n_{\mathrm{total}}}$$

where *n*_triggered_ is the number of the triggered pulses, *n*_total_ the number of the total input pulses. While applying the input pulse with an amplitude of 0.7 V, the real‐time probabilities under various thresholds are shown in Figure 2d. As the threshold increases from 0.5 to 0.68 V, the probability decreases from ≈100% to ≈0%. Thus, the probability of triggered pulses can be modulated in a wide range by adjusting the threshold.

The tunable probability of the triggered pulses was utilized to build dropout neuronal units, as shown in Figure 2e. Comparing with standard neuronal units that are connected to each other through synapses, a dropout neuronal unit is connected to another one through a memristive synapse and an extra probably turned‐on switch. The analog–digital (AD) module converts the received analog signals to digital signals for later processing, and the digital–analog (DA) module is used to generate spikes for signal transmitting. Comparing with the standard neuronal unit, in which the DA module transmits spikes directly, the DA module in the dropout neuronal unit transmits spikes to the synapse through the probably turned‐on switch. Whether the switch is on or off in one operation depends on the output of the comparator: if a pulse is outputted from the comparator, the switch is turned on, and the neuronal unit is activated; if no pulse is outputted from the comparator, the switch is turned off, and the neuronal unit is dropped out. The probability of turning the switch on/off is controlled by the set threshold, in other words, the dropout probability of the neuronal unit can be modulated. Once the operation is over, the stimulation to the stochastic memristive device is cut off. Accordingly, the memristive device relaxes to the HRS automatically without any RESET operation, and the neuronal unit is suppressed.

The dropout neuronal unit with tunable probability can be utilized in different processes of a neural network. In the training process, the unit is activated randomly according to a fixed probability (chosen from 20% to 100% in the later simulation); in the testing process, the unit is always activated (100% activation probability).

*Origins of Volatility and Stochastics*: To better understand the physical origin of these novel performances, we characterized the evolution of the device microstructure after 10 000 switching. A control sample, Ag/Ta_2_O_5_/Pt, was measured for comparison. Furthermore, kinetic Monte Carlo simulations were built to understand the growing filament morphology during electrical stimulation.

The cross‐section TEM image of the Ag/Ta_2_O_5_:Ag/Pt device after 10 000 switching is shown in **Figure** **3**a. Compared with the as‐fabricated sample shown in Figure 1b, the edge between the Ag electrode and the Ta_2_O_5_:Ag layer becomes unclear. And Ag_2_O (Ag^+^) and metallic Ag are found in the Ta_2_O_5_:Ag layer according to the fast Fourier transform (FFT) of the selected area (inset of Figure 3a). Moreover, two crystal clusters are observed inside the Ta_2_O_5_:Ag layer in the high resolution TEM image shown in Figure 3b. Two clusters are separated with a gap, and they are connected with the top electrode and the bottom electrode, respectively. This result is consistent with the previous work about the diffusive memristor based on the Ag filament.^[^ ^39^ ^]^ Based on the above experimental results, it is proposed that Ag in the top electrode migrates into the oxide layer under the electrical field, forming the filament for switching. And the filament diffuses and rupture spontaneously, resulting in the volatile resistive switching.

**Figure 3:**
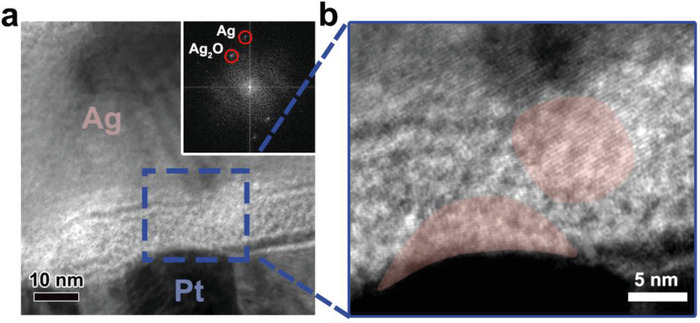
Mechanisms for the volatility and the stochastic switching behavior in the Ag/Ta_2_O_5_:Ag/Pt device. a) TEM image of the device cross‐section after 10^4^ switches. The edge between the Ag electrode and the Ta_2_O_5_ layer becomes unclear, and Ag clusters appear in the Ta_2_O_5_ layer and the edge between the Pt electrode and the Ta_2_O_5_ layer. The inset shows the FFT of the area in the blue dashed rectangle, showing Ag (004) and Ag_2_O (110) in the Ta_2_O_5_ layer. b) Zoom‐in image of the area marked in the dash line in panel (a), showing conducting Ag filaments, as marked by red shadows.

Furthermore, the switching behavior of the control sample of Ag/Ta_2_O_5_/Pt was tested, and the results are shown in Figure S7 in the Supporting Information. It switches to the LRS much slowly than the Ag/Ta_2_O_5_:Ag/Pt device does, and shows much higher LRS currents. More importantly, it takes much longer time (≈300 ms) for the device to recover from the LRS to the original state after removing the electrical pulse. A pulse train consisting of 24 identical pulses was applied to the Ag/Ta_2_O_5_/Pt device, and the device response was recorded in real‐time, as shown in Figure S7b (Supporting Information). Different from the stochastic performance of the Ag/Ta_2_O_5_:Ag/Pt device (Figure 2a; Figures S3 and S4, Supporting Information), the LRS currents of the Ag/Ta_2_O_5_/Pt device are quite similar. The performance differences clearly tell us that the Ag additive accelerates the switching/relaxing speed and enhances the stochastic performance.

To clarify the role of the Ag additive in the switching process, kinetic Monte Carlo simulations of the Ag filament formation were conducted. The simulation was done based on the ion movement or the redox reaction, as shown in Figure S8 in the Supporting Information. First, the electrical potential of each point was calculated by solving the continuity equation via the electrical resistance distribution of the insulating Ta_2_O_5_ and the conducting Ag point. Ag atoms might be oxidized, and Ag^+^ ions might be reduced or moved. The probabilities of the ion movement or the redox reaction were based on the electrical potential.^[^ ^40^, ^41^ ^]^ Finally, an Ag filament was formed, and the device was switched to the LRS. More details about the kinetic Monte Carlo simulation is given in the kinetic Monte Carlo simulation, Supporting Information. The simulated LRS conductance distribution of the Ag/Ta_2_O_5_:Ag/Pt and Ag/Ta_2_O_5_/Pt devices are shown in **Figure** **4**a. The Ag/Ta_2_O_5_:Ag/Pt device shows wider conductance distribution and relatively low conductance compared with those of the Ag/Ta_2_O_5_/Pt device, which is consistent with the above experimental results. The simulated filament morphology and corresponding electrical potential in the Ag/Ta_2_O_5_:Ag/Pt and Ag/Ta_2_O_5_/Pt devices at the LRS state are shown in Figure 4b,c, respectively. The finest part of the filament in the Ag/Ta_2_O_5_:Ag/Pt device is much smaller than the one in the control sample. Since both the volatile speed and the LRS conductance depend on the finest part of filaments,^[^ ^42^, ^43^, ^44^ ^]^ it is reasonable that the Ag/Ta_2_O_5_:Ag/Pt device shows faster volatility and lower LRS currents. The filament in the Ag/Ta_2_O_5_:Ag/Pt device is much weaker.

**Figure 4:**
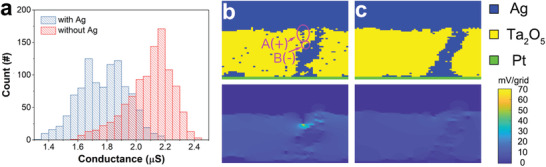
Simulation of the stochastic origination observed in the Ag/Ta_2_O_5_:Ag/Pt device. a) Simulated conductance distribution for the LRS of the Ag/Ta_2_O_5_:Ag/Pt and the Ag/Ta_2_O_5_/Pt devices. b) Simulated filament morphology and electrical potential difference for the Ag/Ta_2_O_5_:Ag/Pt device in the LRS. c) Simulated filament morphology and electrical potential difference for the Ag/Ta_2_O_5_/Pt device in the LRS. The electrical potential of the cluster A is higher than the one at the point B in the filament, forming an effective anode (+) of cluster A and an effective cathode (−) of point B. Therefore, Ag^+^ ions tend to be generated from cluster A and reduced at point B. The process accelerates redox reactions between Ag and Ag^+^.

Under the applied electric field, Ag nanoclusters and filament in the Ag/Ta_2_O_5_:Ag/Pt device can be regarded as bipolar electrodes,^[^ ^45^ ^]^ according to Figure 4b; but similar effects are hardly observed in the Ag/Ta_2_O_5_/Pt device, as shown in Figure 4c. For instance, the potential of the cluster A is higher than the that of the point B in the filament, forming an effective anode (+) of the cluster A and an effective cathode (−) of the point B. Therefore, Ag^+^ ions tend to be generated from the cluster A and reduced at the point B.^[^ ^46^ ^]^ The electrochemical process of bipolar electrodes needs electrons for the cluster A and the point B to satisfy the charge neutrality. The origination of electrons includes the escape of trapped ones, reduction of another Ag^+^, or from the extra reaction (such as the reduction of moisture from the ambient).^[^ ^45^, ^47^, ^48^ ^]^ In addition, Ag nanoclusters with various sizes cause additional surface energy difference, generating extra chemical potential gradients according to the Gibbs–Thomson effect.^[^ ^49^ ^]^ As a result, distributed Ag nanoclusters generate bipolar electrodes with each other. Since bipolar electrodes accelerate redox reactions, it is much easier to change the morphology of the filament and the distribution of the Ag nanoclusters in the Ag/Ta_2_O_5_:Ag/Pt device, which results in the stochastic LRS conductance. Moreover, the acceleration of redox reactions induced by the bipolar electrodes and the Gibbs–Thomson effect leads to the faster volatile speed in the Ag/Ta_2_O_5_:Ag/Pt device as well.

*Dropout Neural Network for Classifying Modified National Institute of Standards and Technology (MNIST) Handwritten Digits*: A three‐layer neural network for classifying handwritten digits (ten digits of 0–9) was constructed based on our dropout neuronal units. As shown in **Figure** **5**a, the neural network has 785 input neuronal units, 151 hidden neuronal units and 10 output neuron units, and every two neuronal units are connected through a synapse. When a testing sample is inputted to the neural network, 10 output neuronal units show different responses. The neuronal unit with the highest response indicates the classification result.

**Figure 5:**
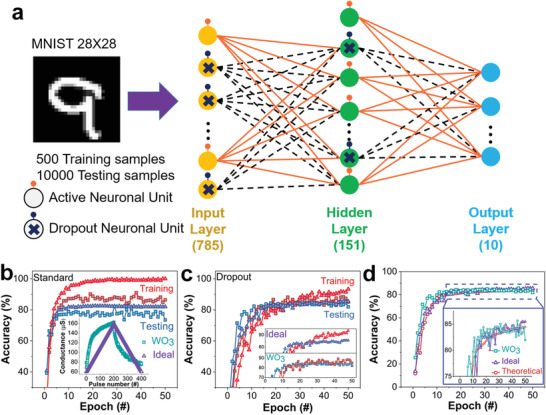
Dropout neural network for the classification of MNIST handwritten digits. a) Schematic illustration of the dropout neural network. b) Accuracy of the neural network without dropout units for training and testing samples, two types of artificial synapses, i.e., ideal and WO_3_‐based ones, are used in the neural network. c) Accuracy of the dropout neural network (50% neuronal units are dropout) with ideal and WO_3_‐based synapses for the testing and training samples. The up panel of the inset shows the magnified accuracy for the ideal synapses, and the low panel of the inset shows the magnified accuracy for the WO_3_‐based synapses. d) Calculated theoretical accuracy of the dropout neural network; data of the real WO_3_ synapses and the ideal synapses are plotted for comparison.

The dropout neuronal units (marked with “×”) are used in the input and hidden layers. Two types of synapses are applied in the network, to test the tolerance of the dropout neurons to nonideality of the connected synapses: one is a Pd/W/WO_3_/Pd memristive device that we prepared (more information is presented in Figure S10 in the Supporting Information), performing nonlinear and asymmetrical conductance modulation; the other one is an ideal memristor with ideally linear and symmetrical conductance change. The analog performances of the memristive synapses in the network are shown in the inset of Figure 5b. To address the Manhattan rule, the synaptic weights are all modulated by applying identical potentiating or depressing pulses.

In the simulation, 500 randomly chosen digits from the MNIST database, a commonly used database for image process systems, are regarded as a small number of training samples to produce the overfitting phenomenon, while all 10 000 testing samples in the database are used to test the accuracy. In one training process, some units (marked with “×” in Figure 5a) are probably dropped out, and their connection to other units are cut off. Thus, a “thinned” network is induced, and the backpropagation algorithm is run for the “thinned” network in an epoch. In the next training process, different neuronal units are activated according to the stochastic performances and a new “thinned” network is trained. After several training process, the unthinned network, i.e., all neuronal units are activated for testing, is able to classify the handwritten digits by comparing the responses of the output neurons. The unthinned network averages the prediction from all “thinned” networks, therefore, it reduces the unexpected features and solves the overfitting problem (more details are presented in Section S6 and Figure S11 in the Supporting Information).

The classification accuracy of the networks based on the WO_3_‐based and ideal synapses are shown in Figure 5b. The square and triangle symbols represent the accuracy based on the WO_3_‐based synapses and the ideal synapses, respectively. The accuracy for the testing samples are much lower than the accuracy for the training samples in both cases, clearly indicating that the overfitting occurs. Moreover, the classification accuracy of the network based on the ideal synapses (≈82%) is higher than the one based on the real WO_3_ synapses (≈78%). The difference demonstrates that the classification accuracy is highly related to the ideality of the synaptic devices. Therefore, the nonideality of the synaptic devices and the lack of sufficient number of samples suppress the classification accuracy of neural networks.

The utilization of the dropout neuronal units helps to reduce the burden of the synaptic performances and addresses the overfitting issue at the same time. When ≈50% neuronal units are dropped out during training, the classification accuracy of the dropout neural network is shown in Figure 5c. First, the network with the dropout neuronal units, either based on the ideal synapses or the real WO_3_ synapses, exhibit comparable classification accuracies to the testing and training samples, and the accuracies are higher than those in Figure 5b. Second, the accuracy difference becomes quite small between the WO_3_‐synapse‐based and ideal‐synapse‐based networks through introducing the dropout neuronal units. The theoretical accuracy level of the neural network, as calculated by the mature software algorithm, is given in Figure 5d. Notably, the accuracy of the dropout neural network based on the real synapses is very close to the theoretical limit in the classification of a small number of samples. These results clearly demonstrate that the error coming from the nonideality of synapses can be compensated through introducing the dropout neural units.

In iteration, to add the dropout function to the neuronal units improves the classification accuracy in the case of insufficient number of training samples by suppressing the overfitting, and reducing the requirements for memristive synapses. Actually, the overfitting is induced by the mistake record of wrong features. In each training process with a small number of training samples, differently “thinned” networks record different features, including right features and wrong ones, i.e., noise. Apparently, the wrong features are recorded with lower probability. When the “thinned” networks are combined, the wrong features are diluted, and the overfitting issue is settled.

Nonidealities of the synapses, i.e., nonlinearity and asymmetry in the conductance modulation, are considered as the updating noise, resulting in the accumulation of the error during frequent updating in training processes. Thus, reducing the unnecessary updating helps to mitigate the negative effect of the synaptic nonideality.^[^ ^50^ ^]^ Similarly, the updating frequency is decreased by probably dropping out some neuronal units. As a result, the error from the nonideality can be decreased. In other words, the negative effect of the nonideality of the synapses on the classification accuracy is suppressed. The accuracies of the networks with different dropout probabilities verify it, as shown in **Figure** **6**; increasing the dropout probability decreases the difference between the WO_3_‐synapse‐based and the ideal‐synapse‐based networks.

**Figure 6:**
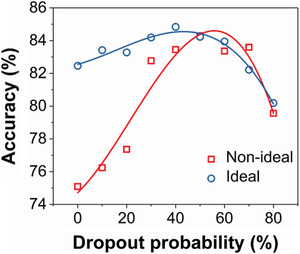
Relationship between the accuracy and the dropout probability. As the dropout probability increases, the difference between the networks based on ideal synapses and nonideal synapses are mitigated.

In this work, we demonstrate the dropout neuronal unit based on the stochastic volatile memristive device of Ag/Ta_2_O_5_:Ag/Pt, which is probably dropped out in the training process. The memristive neural network utilizing the dropout neuronal units shows a high accuracy in classifying handwritten digits by suppressing the overfitting in the case of insufficient number of training samples, and mitigating the error from the nonideality of the memristive synapses. The physical nature of the increased stochastics and accelerated volatility of the device is investigated by the TEM observation and the kinetic Monte Carlo simulation, and attributed to the additive of Ag in the Ta_2_O_5_ layer. The implementation of the dropout neuronal units promotes the application of memristive neural networks in the face of limited training samples and the absence of ideal artificial synapses. Although the function of the dropout neuronal units is demonstrated with the recognition of the MNIST handwritten‐digit database, this approach can be extended to other neuromorphic systems based on memristive devices to boost their performance.

## Experimental Section

##### Sample Preparation

Ag/Ta_2_O_5_:Ag/Pt memristive devices were fabricated on silicon wafers with 500 nm thermally grown SiO_2_. First, 100 nm thick Pt bottom electrodes, with 5 nm Ti adhesive layer, were deposited on the wafer by DC sputtering (Angstrom Engineering Magnetron Sputtering System). Second, 12 nm thick Ta_2_O_5_:Ag layers were prepared by cosputtering with a Ta_2_O_5_ and a Ag target. Finally, Ag top electrodes were deposited by DC sputtering. The three layers were all prepared in Ar, and lift‐off was applied in every step.

##### Structure Characterizations and Electrical Property Measurements

A transmission electron microscope (TEM, Netherlands Tencai G2 F30) with EDX was employed to characterize the microstructure evolution of the device during electrical stimulation. TEM samples were prepared by means of focused ion beam (FIB, Helios NanoLab G3 CX). All the electrical measurements were conducted with a Keithley 4200 SCS connected with a Cascade SUMMIT 11000B semiautomatic probe station and a Keysight DSOX3104T oscilloscope in air at room temperature. The positive voltage was applied to the Ag top electrode, while the Pt bottom electrode was grounded.

## Conflict of Interest

The authors declare no conflict of interest.

## Supporting information

Supporting Information
