# Supplementary material for: Implementation of Dropout Neuronal Units Based on Stochastic Memristive Devices in Neural Networks with High Classification Accuracy
Source: Adv Sci (Weinh). 2020 Jul 26;7(18):2001842. doi: 10.1002/advs.202001842 (PMC7509653; doi:10.1002/advs.202001842)
Supplement: Supplementary file 1 — Supporting Information [file ADVS-7-2001842-s001.pdf]

Copyright WILEY-VCH Verlag GmbH & Co. KGaA, 69469 Weinheim, Germany, 2020.

ADVANCED SCIENCE
Open Access

Supporting Information

for *Adv. Sci.*, DOI: 10.1002/advs.202001842

Implementation of Dropout Neuronal Units Based on Stochastic Memristive Devices in Neural Networks with High Classification Accuracy

He-Ming Huang, Yu Xiao, Rui Yang, Ye-Tian Yu, Hui-Kai He, Zhe Wang, and Xin Guo**

Copyright WILEY-VCH Verlag GmbH & Co. KGaA, 69469 Weinheim, Germany, 2020.

Supporting Information

Implementation of Dropout Neuronal Units Based on Stochastic Memristive Devices in Neural Networks with High Classification Accuracy

He-Ming Huang, Yu Xiao, Rui Yang, Ye-Tian Yu, Hui-Kai He, Zhe Wang, Xin Guo**

1. Measurement of Ag content in the Ta_2O_5 layer

EDX line scanning was employed to check the Ag content in the Ta_2O_5 layer, results are shown in Figure S1. Ag and Ta elements coexist in the layer between the Pt and Ag electrodes, indicating that Ag diffuses into the Ta_2O_5 layer. We used XPS to measure the Ag content, results are shown in Figure S2 and Table S1. The atomic ratio of Ag in the Ta_2O_5 layer is calculated to be ~3%.

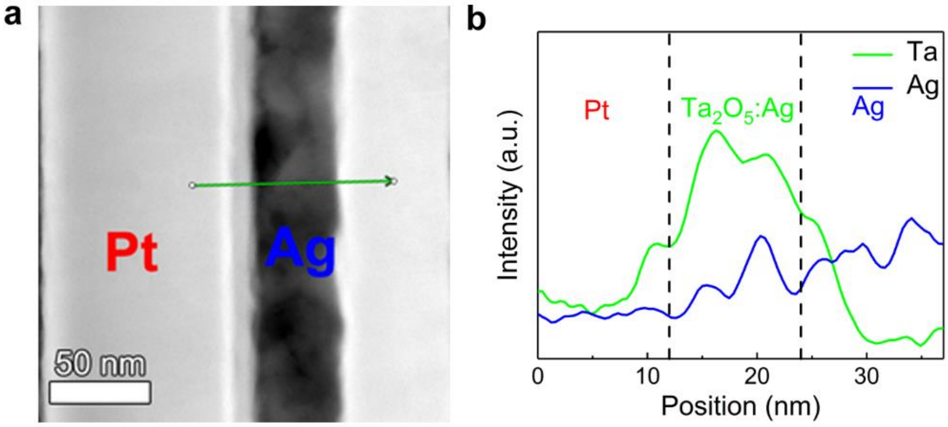

Figure S1 (a) TEM image and (b) EDX of line scanning of the Ag/Ta_2O_5:Ag/Pt device.

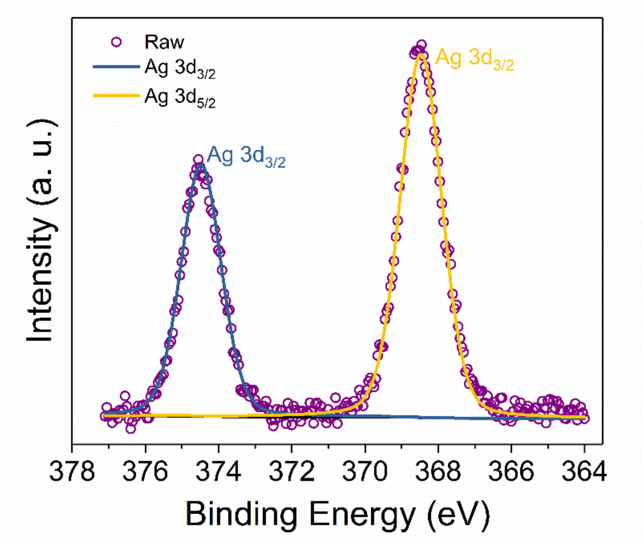

Figure S2 XPS spectrum of Ta_2O_5:Ag.

Table S1 Element concentrations as determined from XPS

Peak	Position (eV)	FWHM (eV)	Raw Area	RSF	Atomic concentration (%)
O 1s	529.80	2.623	40053.3	0.780	73
Ag 3d	367.35	1.243	10933.5	5.987	3
Ta 4f	26.76	1.478	53699.63	3.080	24

2. Stochastic performances of the Ag/Ta_2O_5:Ag/Pt device

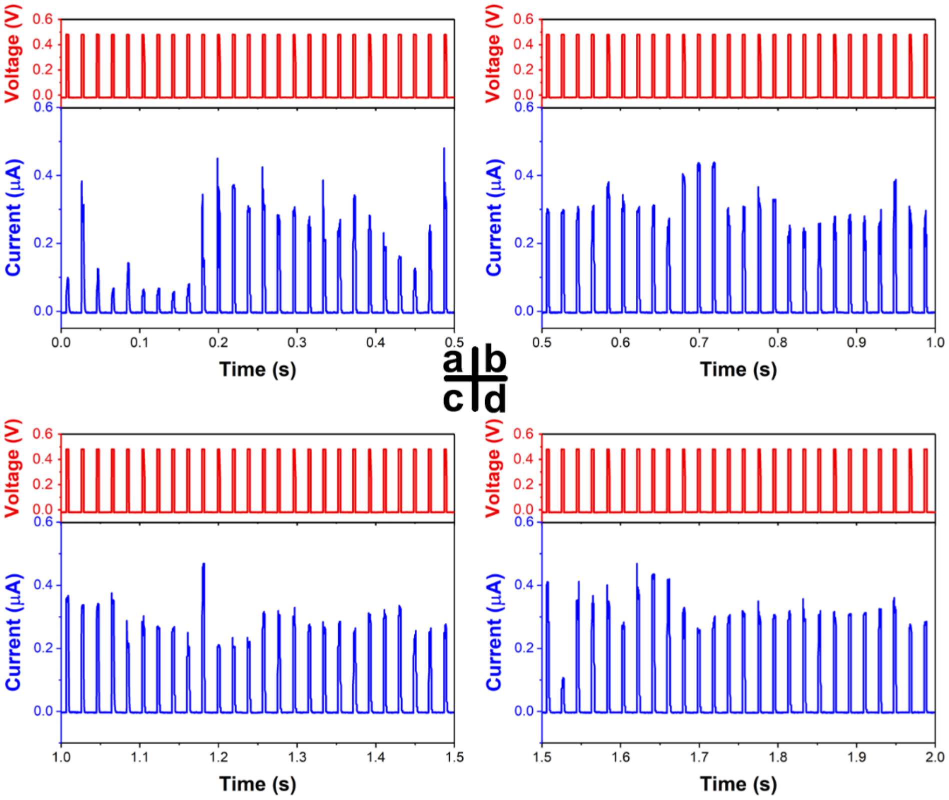

Figure S3 Different responses of the Ag/Ta_2O_5:Ag/Pt device to100 identical electrical pulses.

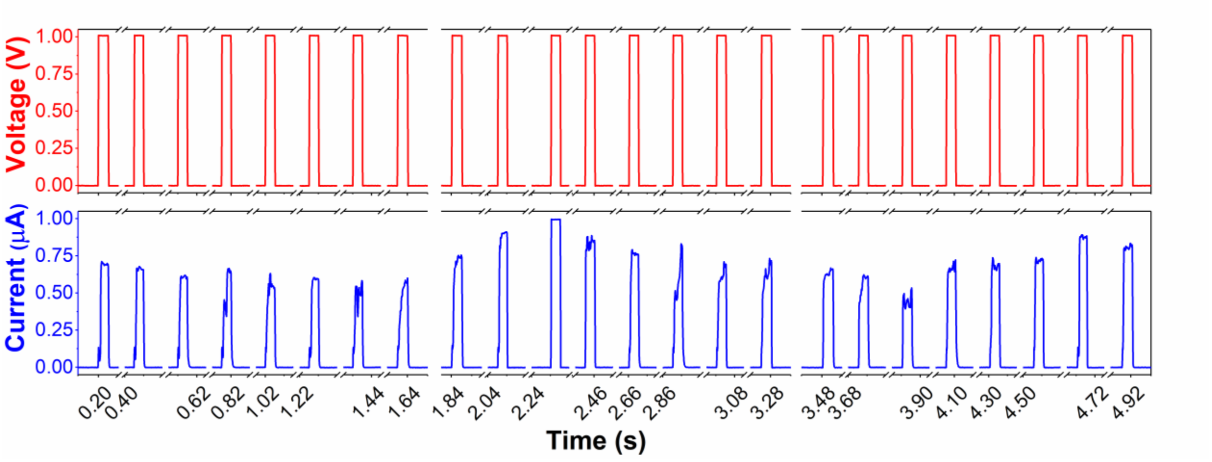

Figure S4 Responses of the Ag/Ta_2O_5:Ag/Pt device to 24 identical pulses of 1.0 V, showing stochastic currents.

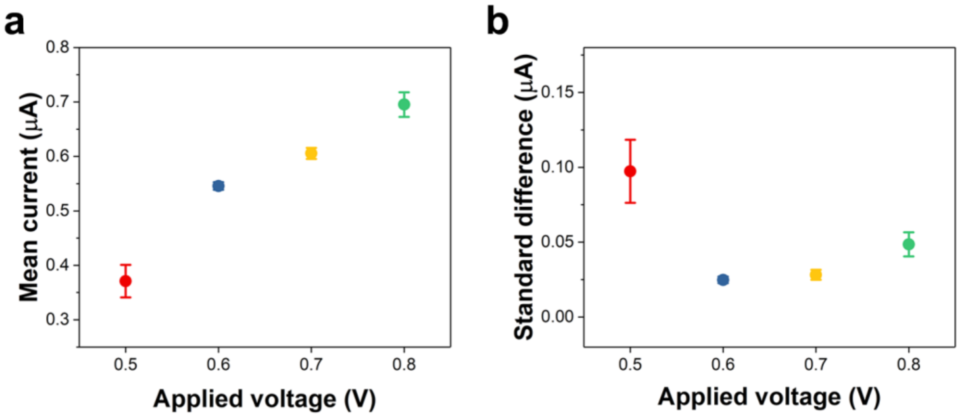

Figure S5 Cycle-to-cycle variation of the response currents of the Ag/Ta_2O_5:Ag/Pt device: a) Mean currents under various applied voltages; b) Standard differences of the response currents under various applied voltages.

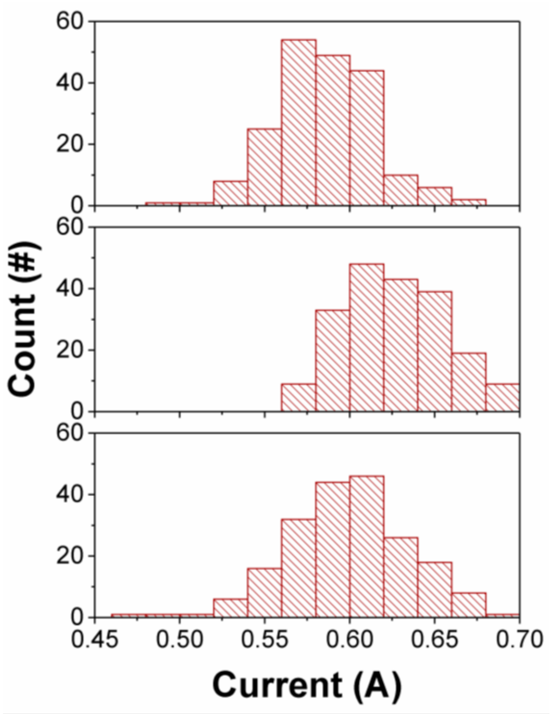

Figure S6 Distribution of the response currents of three devices to the pulse of 0.7 V.

3. Performance of the control sample

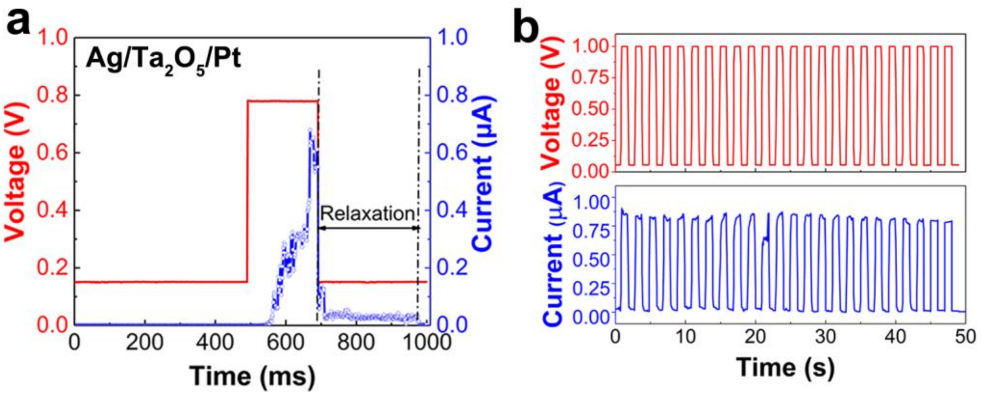

Figure S7 Electric performances of the Ag/Ta_2O_5/Pt device. a) Pulse response of the Ag/Ta_2O_5/Pt device, the relaxation time is much longer than that of the Ag/Ta_2O_5:Ag/Pt device. b) Responses of the Ag/Ta_2O_5/Pt device to 24 identical electric pulses, showing similar response currents without stochastic feature.

4. Kinetic Monte Carlo simulation

i. Modelling

Possible events are described in the Monte Carlo simulation shown in Figure S8: (a) oxidation, (b) surface migration, (c) desorption, (d) migration in the insulating matrix, (e) adsorption, (f) reduction and (g) nucleation.[S1]

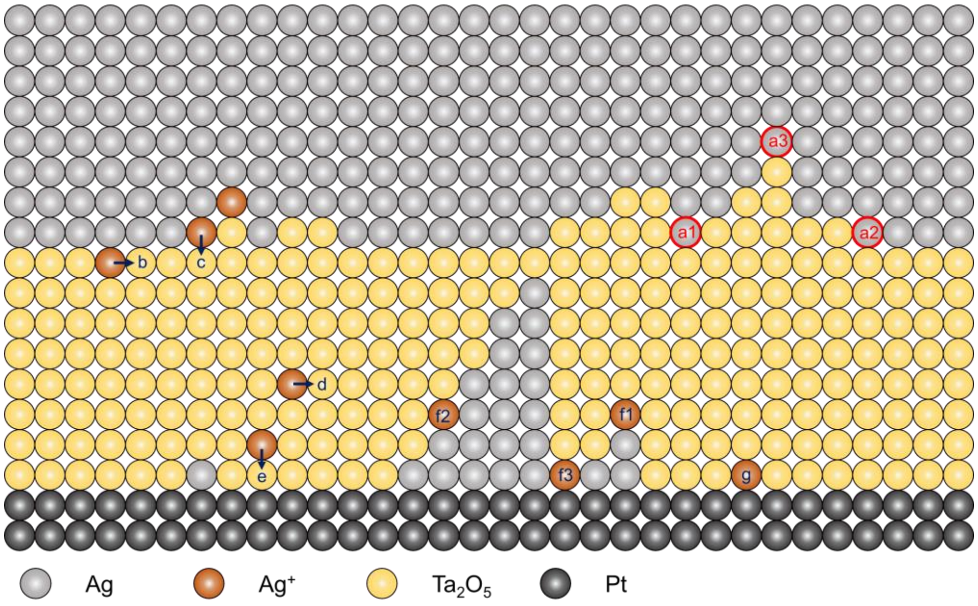

Figure S8 Possible events involved in the switching: oxidation at adatom site (a1), kink site (a2), and hole site (a3); surface migration (b); desorption (c); bulk migration (d); adsorption (e); reduction at adatom site (f1), kink site (f2) and hole site (f3); nucleation (g).

The transition rate Γ of each event can be calculated by:

(a) oxidation

$$\Gamma_{ox} = \nu_0 \exp\left(-\frac{\Delta W_{ox,state} + (1-\alpha)zeV}{k_B T}\right) \tag{S1}$$

where ν_0 is the phonon frequency, $\Delta W_{ox,state}$ the activation energy for the oxidation in various states (adatom, kink and hole site), α the charge transfer coefficient, z the ion charge number, e the elementary charge, k_B the Boltzmann constant and T the local temperature.

(b) reduction

$$\Gamma_{re} = \nu_0 \exp\left(-\frac{\Delta W_{re,state} - \alpha zeV}{k_{\mathrm{B}}T} \right) \tag{S2}$$

where $\Delta W_{re,state}$ is the activation energy for the reduction in various states (adatom, kink, hole site and nucleation).

(c) migration

$$\Gamma_m = \nu_0 \exp\left(-\frac{\Delta W_{m,state} - 1/2 ze\Delta\varphi}{k_{\mathrm{B}}T} \right) \tag{S3}$$

where $\Delta W_{m,state}$ is the activation energy for the migration in various states (surface, bulk, desorption and adsorption).

Table S2 Parameters for simulation

Symbol	Value	Symbol	Value
ν_0	1×10^{13} Hz	$\Delta W_{re,adatom}$	0.51 eV
h	6.63×10^{-34} J·s	$\Delta W_{re,kink}$	0.48 eV
α	0.5	$\Delta W_{re,hole}$	0.46 eV
z	1	$\Delta W_{re,nucleation}$	0.60 eV
T	300 K	$\Delta W_{m,surface}$	0.36 eV
m_{eff}	1.6×10^{-19} kg	$\Delta W_{m,bulk}$	0.40 eV
$\Delta W_{ox,adatom}$	0.53 eV	$\Delta W_{m,desorption}$	0.41 eV
$\Delta W_{ox,kink}$	0.55 eV	$\Delta W_{m,adsorption}$	0.35 eV
$\Delta W_{ox,hole}$	0.58 eV	ΔW_0	4.00 eV

Based on the transition rate, the cumulative probability R_i of each event can be calculated by:

$$R_i = R_{i-1} + \frac{\Gamma_i}{\sum_t \Gamma_t} \tag{S4}$$

Parameters used in the simulation are listed in Table S2.

ii. Calculation of the electric potential

To calculate the probability of each process, the electric potential in the device is determined by solving the current continuity equation with the finite-difference method:

$$-\nabla \sigma \nabla \varphi = 0 \tag{S5}$$

where φ and σ are the electric potential and the conductance of a specific position, respectively. When a nanocluster approaches another one, the tunnel contact sets in although they do not physically contact. The tunnel current density J_{TC} is calculated according to the linear Simmons equation:

$$J_{TC} = \frac{3\sqrt{2m_{eff}\Delta W_0}}{2x}\left(\frac{e}{h}\right)^2 \exp\left(-\frac{4\pi x}{h}\sqrt{2m_{eff}\Delta W_0}\right)V \tag{S6}$$

where m_{eff} is the effective electron mass, ΔW_0 the tunneling barrier height, x the tunneling distance, and h the Planck's constant. Notably, a resistor is added in series to compile the maximum current, so the current is calculated according to:

$$R_{read} = \frac{V_{read}}{\sum_i \sigma_{y,i} \nabla \varphi_{y,i|read}} \tag{S7}$$

$$I = \frac{V_{input}}{R_{read} \,||\, R_s} \tag{S8}$$

where i means the ith atom in a row, σ_y and φ_y are the conductance and the electric potential difference in the column, and R_s is the resistor in series.

iii. Simulation procedure

The simulation is performed according to the flowchart shown in Figure S9. The initialization

is firstly performed, then the electric potential of each grid point is calculated according to the continuity equation, and the event transition rates are calculated based on the potential. The transition rates are summed up to get the occurring probability of each event.

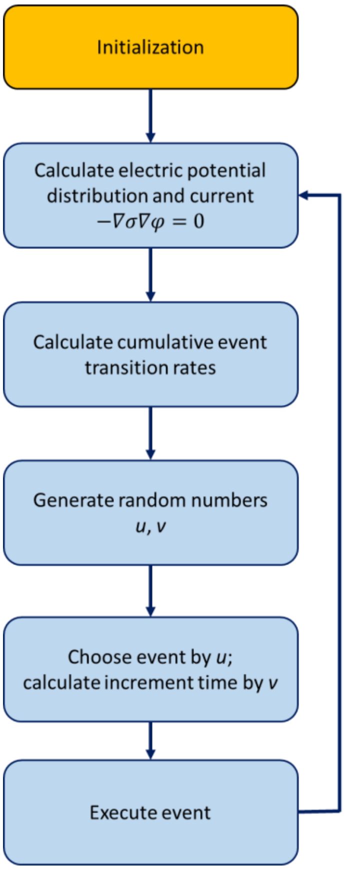

Figure S9 Flowchart of the Monte Carlo simulation for the generation of filament.

Later, two random number u and v are generated for choosing event and calculating increment time, respectively. When $R_i \leq uR_N \leq R_{i+1}$, the event i is chosen, then the using time Δt is updated by:

$$\Delta t = -\log(v) / R_N \tag{S9}$$

Finally, the chosen event is executed and go to the next cycle.

5. Structure and performance of the Pd/W/WO_3/Pd device

Pd/W/WO_3/Pd memristive devices were fabricated on silicon wafers with 500 nm thermally grown SiO_2. First, 80-nm-thick Pd bottom electrodes were deposited on the wafer by DC sputtering. Second, 80-nm-thick WO_3 layers were prepared by RF sputtering with a WO_3 target. Finally, 20 nm Pd and 80 nm W top electrodes were deposited by DC sputtering. The pattern of the top electrode was prepared by a metal mask.

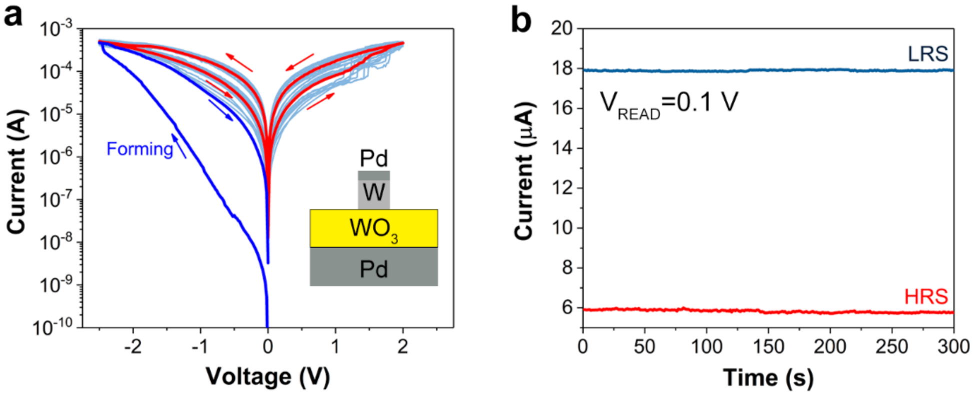

Figure S10 Switching behavior and structure of the Pd/W/WO_3/Pd memristive device. a) Current-voltage curves of the device in electroforming process and sweep loops, the inset is the structure of the device. The device needs a negative electroforming, and then shows the positive SET and the negative RESET. b) Retention performance when applying a 0.1 V READ voltage, showing a nonvolatile performance. Positive or negative voltages were applied to the Pd/W top electrode, while the Pd bottom electrode was grounded

6. Simulation of the dropout neural network

Figure S11 shows the flowchart of the simulation. Firstly, the conductance of the devices in the networks are set randomly, and the weights are represented by the difference between the conductance of the device and a resistor. Secondly, control pulses are generated to the dropout neuronal units and parts of the neuronal units are activated. Thirdly, training data, including 500 handwritten digits, forward propagates to the network with five mini-batches.

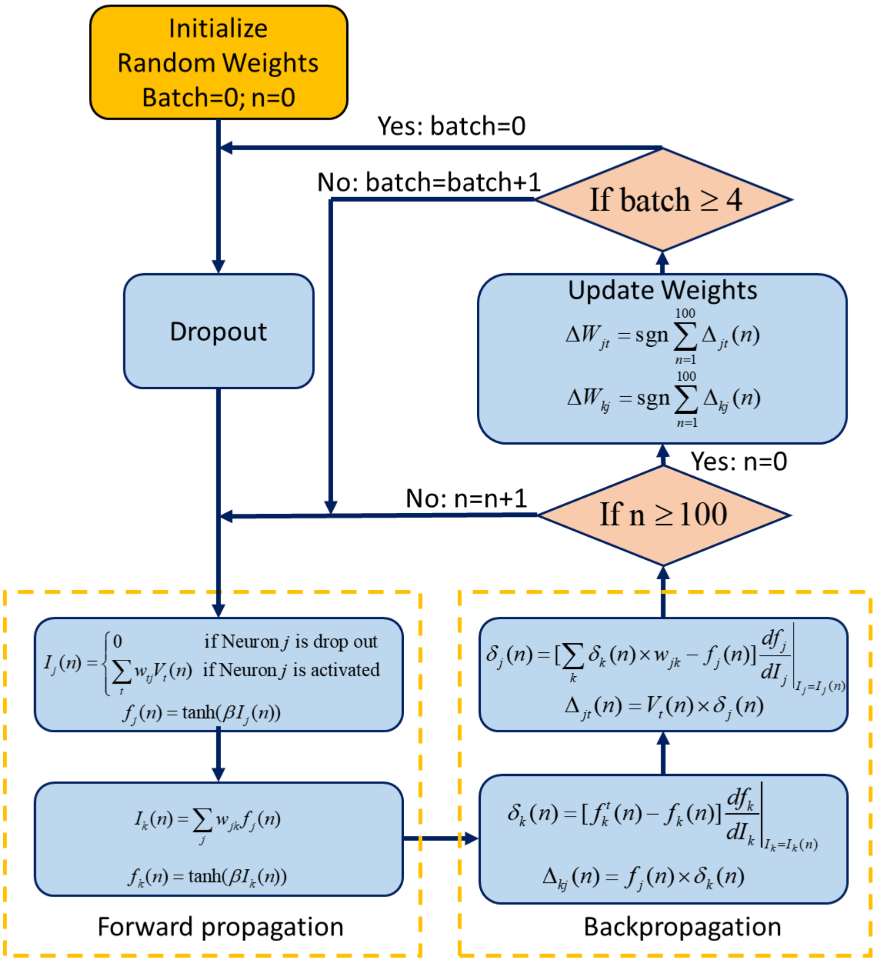

Figure S11 Flowchart for the simulation of the neural network based on dropout neuronal units.

100 samples are inputted in each batch according to Equations S10-S12:

$$I_j(n) = \begin{cases} 0 & \text{if Neuron } j \text{ is drop out} \\ \sum_t w_{tj} V_t(n) & \text{if Neuron } j \text{ is activated} \end{cases} \tag{S10}$$

where I_j is the input of the Neuron j in the hidden layer, w_{jt} the weight of the synapse connected to the Neuron j, and V_t represents the related pixel t in the pattern.

$$f_j(n) = \tanh(\beta I_j(n)) \tag{S11}$$

where f_j is the output of the Neuron j, and β the coefficient for the specific range of the device conductance.

$$I_k(n) = \sum_j w_{jk} f_j(n) \tag{S12}$$

where I_k is the output current of the Neuron k in the output layer, w_{jk} the weight of the synapse connected to the Neuron k. The neuron unit with the highest output current is the classification result.

Then error is back propagated for gradient descent to calculate the weight updating according to Equations S13-S19:

$$f_k(n) = \tanh(\beta I_k(n)) \tag{S13}$$

$$\delta_k(n) = [f_k^t(n) - f_k(n)] \left. \frac{df_k}{dI_k} \right|_{I_k = I_k(n)} \tag{S14}$$

$$\Delta_{kj}(n) = f_j(n) \times \delta_k(n) \tag{S15}$$

$$\delta_j(n) = [\sum_k \delta_k(n) \times w_{jk} - f_j(n)] \left. \frac{df_j}{dI_j} \right|_{I_j = I_j(n)} \tag{S16}$$

$$\Delta_{jt}(n) = V_t(n) \times \delta_j(n) \tag{S17}$$

where $\delta_k(n)$, $f_k^t(n)$ are the error and the expectation for the Neuron k, $\Delta_{kj}(n)$, $\Delta_{jt}(n)$ the updating weights for the synapses w_{kj} and w_{jt} with the input of n, respectively.

After all the training samples in one mini-batch are inputted, the weight changes are summed up for updating according to the Manhattan rule:

$$\Delta W_{jt} = g[w_{jt}, \mathrm{sgn}\sum_{n=1}^{100}\Delta_{jt}(n)] \tag{S18}$$

$$\Delta W_{kj} = g[w_{kj}, \mathrm{sgn}\sum_{n=1}^{100}\Delta_{kj}(n)] \tag{S19}$$

where *g(w, sgn)* is the function of potentiation or depression for memristors. One iteration is over when next four mini-batches are finished.

Reference:

[S1] S. Menzel, P. Kaupmann, R. Waser, *Nanoscale* **2015**, *7*, 12673.
